# Open cavity abdominal surgery in octogenarians and nonagenarians admitted to a university teaching hospital ICU: a retrospective review

**DOI:** 10.1186/cc13385

**Published:** 2014-03-17

**Authors:** A Roberts, P Hampshire

**Affiliations:** 1 Royal Liverpool University Hospital, Liverpool, UK

## Introduction

This review was undertaken to establish the ICU and hospital mortality rates in patients aged 80 and over admitted to our ICU following abdominal cavity surgery. Intensive care mortality increases progressively with age [1] and as the population changes we are likely to see increasing numbers of patients over the age of 80 admitted to critical care units.

## Methods

We searched the ICNARC database from 2006 to 2013 for patients aged 80 years or over, admitted from theatre or after surgery. Data were referenced against the electronic theatre management system, and patients not undergoing abdominal cavity surgery were excluded. The data were analysed using an Excel spreadsheet (Microsoft) and Medcalc software.

## Results

Eighty-five patients were included, with ages ranging from 80 to 99 years. Fifty-one (60%) patients were male and 79 (93%) patients were categorized as having an emergency operation. ICU mortality was 34/85 (40%) and hospital mortality was 48/85 (56%). Variables assessed for association with hospital mortality can be seen in Table 1. Only invasive ventilation and time (days) from hospital admission to ICU admission were significant predictors of hospital mortality.

**Table 1:** Survivors versus nonsurvivors

	Survivors	Nonsurvivors	*P *value
Age	83	83	0.82
Time	1, 1 to 8	4.5, 1 to 8	0.004
APACHE II	17, 14 to 2C	17, 14 to 2'	0.96
Sex (%)	M57, F43	M63, F37	0.65
Ventilation (*n*, %)	31, 83	48, 100	0.005
RRT (*n*, %)	6, 16	11, 23	0.58

Data presented as median, IQR or *n*, %.

## Conclusion

ICU and hospital mortality rates were high at 40% and 56% respectively. Increasing age did not correlate with mortality. However, invasive ventilation and time between hospital and ICU admission were associated with a higher mortality. This may be due to a delay in diagnosis or surgical intervention. We suggest that one considers early intervention in patients aged over 80. A functional outcome measure at discharge may be a more clinically relevant endpoint.
